# Diastolic timed Vibro-Percussion at 50 Hz delivered across a chest wall sized meat barrier enhances clot dissolution and remotely administered Streptokinase effectiveness in an in-vitro model of acute coronary thrombosis

**DOI:** 10.1186/1477-9560-10-23

**Published:** 2012-11-12

**Authors:** Andrew Hoffmann, Harjit Gill

**Affiliations:** 1 Ahof Biophysical Systems Inc, 3858 Regent St, Burnaby, BC, Canada , V5C4G8; 2 In-Vitro Labs, 5407 Canada Way, Burnaby, BC, Canada , V5E 3N5

**Keywords:** Vibration, Thrombolysis, STEMI, Reperfusion, Diastole

## Abstract

**Background:**

Low Frequency Vibro-Percussion (LFVP) assists clearance of thrombi in catheter systems and when applied to the heart and timed to diastole is known to enhance coronary flow. However LFVP on a clotted coronary like vessel given engagement over a chest wall sized barrier (to resemble non-invasive heart attack therapy) requires study.

**Methods:**

One hour old clots (n=16) were dispensed within a flexible segment of Soft-Flo catheter (4 mm lumen), weighted, interfaced with Heparinized Saline (HS), secured atop a curved dampening base, and photographed. A ~4 cm meat slab was placed over the segment and randomized to receive intermittent LFVP (engaged, - disengaged at 1 second intervals), or no LFVP for 20 minutes. HS was pulsed (~120/80 mmHg), with the diastolic phase coordinated to match LFVP delivery. The segment was then re-photographed and aspirated of fluid to determine post clot weight. The trial was then repeated with 0.5 mls of Streptokinase (15,000 IU/100 microlitre) delivered ~ 2 cm upstream from the clot.

**Results:**

LFVP - HS only samples (vs. controls) showed; a) development of clot length fluid channels absent in the control group (p < 0.0002); b) enhanced dissolved clot mixing scores ( 5.0 vs. 0.8, p < 2.8 E – 6); and c) increased percent clot dissolution (23.0% vs. 1.8% respectively, p < 8.5 E-6). LFVP - SK samples had a similar comparative clot disruptive profile, however fluid channels developed faster and percent clot dissolution more than doubled (51.0% vs. 3.0%, p< 9.8 E- 6).

**Conclusion:**

Diastolic timed LFVP (50 Hz) engaged across a chest wall sized barrier enhances clot disruptive effects to an underlying coronary like system.

## Introduction

Thrombo-occlusive cardiovascular disease is the leading cause of mortality in the developed world [1]. Among these, acute ST-Elevation Myocardial Infarction (STEMI) is an important disease with high morbidity and mortality, of which rapid and complete blood flow restoration to the occluded artery is the main determinant of clinical outcome [2]. Currently STEMI is preferably treated by emergency Primary Percutaneous Coronary Intervention (PPCI) where a balloon and a stent are deployed to open the acutely thrombosed vessel. However significant infrastructure is required for PPCI and there are sometimes lengthy delays (often greater than 90 min) in delivering a patient which leads to increased mortality and morbidity [3]. Intravenous (IV) thrombolytic therapy therefore remains a common alternative therapy worldwide particularly in rural areas [4], however IV thrombolysis has a relatively low complete reperfusion rate and has worrisome potential hemorrhagic complications [5]. There is therefore an ongoing search for non-invasive therapies with less serious side effects to enhance early restoration of coronary flow.

Transthoracic ultrasound (involving imperceptible high frequency vibrations above 20 kHz) has been investigated to improve IV thrombolysis in STEMI, however clinical trials involving this technique (PLUS Trial [6]), have thus far failed to show efficacy, probably due to the problematic high attenuation characteristics of ultrasound through a relatively thick chest wall and lung [7], with thereby an apparent need for advanced skill based imaging techniques to direct the therapy [8]. Ultrasound further harbors a propensity, especially at higher intensities, to lead to increased platelet adhesion [9], damage blood vessels [10-15] and burn the overlying skin of patients receiving therapy [13,14].

Our group has been working on the premise that infrasonic to sonic Low Frequency Vibro-Percussion (LFVP) (a waveform with a much higher, *palpable* stroke amplitude than ultrasound), may offer a safe, effective acoustic alternative in the non-invasive treatment of STEMI due to LFVP’s known clot disruptive abilities [16-20], superior deep penetration capabilities across the chest wall [21,22] and unique internal transmission characteristics through body tissue including arteries and the epi-myocardium [23-26]. However study of this area has been scarce, and there has yet to be even basic in-vitro verification that LFVP if engaged over a substantial tissue attenuation barrier (i.e. sized comparably to a human chest wall) would actually penetrate to provide agitative thrombo-clearing effects to an underlying clotted, coronary like vessel. We have previously reported on LFVP’s ability to clear thrombosis in a coronary like flow tube when applied over a 2 cm thick meat barrier [20], but have been criticized that the chest wall is considerably thicker than 2 cm, and that we did not test LFVP’s effects in combination with its preferred medical adjunct - a thrombolytic agent.

Hence we periodically engaged a 50 Hz, ~4 mm percussive massager against a 4 cm thick meat barrier directly overlying a clot filled, Heparinized, coronary like tube system to assess whether LFVP would penetrate and enhance clot dissolution and/or any other clot disruptive effects within the tube. We then repeated the trial with a co-administration of Streptokinase (SK) administered remotely ~ 2.0 cm upstream from the clot.

## Methods

Work developing our experiment was performed in a private lab setting (In-Vitro Labs, Burnaby, B.C., Canada). The test subjects (the Authors, a healthy male and female), who donated blood gave consent to the study which had been approved by Institutional Review [27] in accordance to the U.S. Department of Health & Human Services Basic Policy for Protection of Human Research Subjects.

### Vibrating equipment

We chose a hand-held massager (Acuvibe HT 1280) which oscillates at 50 Hz with a percussive stroke amplitude of ~4 mm as judged by the width of the contact node’s corona during active vibration. This provided for a moderate intensity vibration which we have shown in our lab to be tolerable when applied to the ribcages of both men and women.

A percussive frequency of 50 Hz was chosen as it has been shown that externally delivered *diastolic* timed LFVP (“dLFVP”) at 50 Hz enhances cardiac performance [21,28,29] as well as coronary flow, both in animals and humans, in the ischemic heart [30-32]. Furthermore, 50 Hz is also within the range of frequencies used in vibrating thrombolysis catheters such as the Trellis® 8 Peripheral Infusion System (Bacchus Medical) which uses motor speeds up to 3500 rpm which causes direct mechanical oscillation of the catheter’s wire [16]. Moreover 50 Hz is in the range of frequencies shown to induce transverse wave propagation along the heart’s epi-myocardium and along the length of arteries, [23-26], hence once applied – and unlike ultrasound - should provide an optimized internalized propogative effect to the entirety of a coronary circulation regardless of overlying acoustic attenuating structures and without a need for skill based targeting of the therapy. Additionally, LFVP including at 50 Hz produces convection currents and vortices in fluids [33,34], which we have observed first hand to be highly desirable in effecting erosion and mixing of blood clot constituents. Finally, to our knowledge there has been no reported evidence of harmful effects of infrasonic to sonic frequency vibration (i.e. for short term therapeutic exposures) in tissue heating or on blood vessels [Medline review, 2012].

It is again emphasized that this trial does not involve the employment of *ultrasound*, but rather a much lower frequency and higher amplitude, palpable, vibratory - percussive waveform in the *low sonic* frequency ranges.

### “Diastolic” timing

As an emergency transthoracic LFVP for STEMI application would foresee ably be timed to turn “on” during diastole and “off” during systole (i.e. “dLFVP”), we mimicked this condition by engaging and then very briefly disengaging our massager at one second intervals timed by a metronome. The administration of pulsated pressure was coordinated by a second investigator such that the “diastolic” (lower phase) of the pulsated waveform matched delivery of LFVP.

### Adjunctive pharmacology

#### Heparinized Saline (HS)

As a clot/fluid interface we chose pressurized Heparinized Saline (HS-1000 Units/500 ml bag in 0.9% NACL @~pulsated ~ 120/80 mm HG, ~ 60 bpm), to ensure clot constituents would not reform once disaggregated, and to provide a small degree of anticoagulant drug type therapy (with pulsatile arterial like filtration-pressure) to closer mimic an anticipated emergency clinical scenario.

#### HS plus Streptokinase (SK)

As an adjunctive thrombolytic agent we chose Streptokinase (75,000 IU ~ 15,000 IU/100 microlitre, 0.5 ml bolus) administered to the proximal end of our thrombosed catheter segment in compliment to the adjoining distal aspect of our connecting line which equated to an interfacing edge SK location (depending on clot length) of approximately 1.5 to 2.5 cm (usually ~ 2.0 cm) upstream from the clot. Remote SK delivery was employed to mimic an intravenous (or systemic) delivered dose of thrombolytic which would initially be of negligible concentration in direct proximity to an acute thrombosis site within a culprit artery. As a stock solution to a commercially available lyophilized SK vial (750,000 I.U.) 5 ml of sterile water was added and mixed to yield a concentration of 15,000 IU/100 microlitre^a^. The stock solution was kept refrigerated at 4 degrees Celsius when not in use (for repeat testing on following days), and was always discarded and replenished after 5 days following reconstitution.

### Harvesting and treatment of clots

The experiment was performed via the assistance of two blood donors (the Authors – male 46 yrs, female 50 yrs), neither having evidence of blood coagulation disorders^b^ or acute illness, and without a recent history of oral contraceptive use and never anticoagulant therapy.

For each sample run an aliquot of blood was drawn to fill a red top vacucontainer which was then held upright within the armpit of an investigator for 1 hour to maintain core temperature. This produced a long columnar “mother” clot which was laid upon a dry plastic sheet and immediately roll dried for 20 cm, allowed to air dry for 5 minutes, and then further roll dried for another 20 cm to rid the clot of gross amounts of adherent serum. We then cut a ~ 0.75 cm piece of “daughter” clot lengthwise, roll dried it for 30 cm, air dried for one minute, and then role dried again for an additional 30 cm. The daughter clot (now “dry” enough of adherent serum to handle) was then corralled into the open end of a 3 ml syringe, where after a pre-weighted segment of Soft Flo catheter (with a 4 mm lumen diameter – a size consistent with a large epicardial coronary artery) was uncapped distally to receive the clot by needleless injection. Once loaded the catheter segment was tilted with the uncapped distal end facing downwards to allow gravity to migrate the delivered clot flush against the distal cap during re-securement. The capped catheter segment was then wiped and re-weighted to obtain a pre-clot weight. Blood clots were manipulated in this manner for each test run.

### Establishment of vibration system

At the beginning of each testing period an HS solution bag spiked with a regular connecting line was placed in a pressure sleeve and hung. Prior to each test run HS was slowly injected into the uncapped proximal aspect of the catheter segment to fill its lumen while not introducing air or significantly agitating the clot within. We then forcefully secured the open blue tipped proximal end of the catheter segment (drop to drop, to yield a fluid connection) with the connecting line. The catheter segment (now with connecting line) was then secured with tape (Transpore, 3M) upon a curved gel pad wrist support (Fellows Inc.), and the HS bag was pressurized to ~80 mm Hg. We then photographed the system to document the clot’s initial condition and how much fluid/clot boundary mixing of dissolved clot constituents had initially taken place (a grade of 0 was given if there was incomplete catheter filling of partially transparent, red color in the lengthwise dimension, a grade of 1 if there was complete catheter filling of red color only in the lengthwise dimension but not at any point in the height wise dimension, a grade of 2 being same as 1 but if any red color had migrated beyond the blue tipped connector of the catheter segment into the connecting line, a grade of 3 if color was at any point fully red within the catheter in its height dimension, a grade of 4 if the catheter became completely red - height and length - with up to 2 cm of color having permeated into the connecting line, a grade of 5 being the same as 4 but between 2 cm and 20 cm of red color having permeated proximally into the connecting line, a grade of 6 being same as 5 but with red color having permeated beyond 20 cm and up to 30 cm up into the connecting line, and finally a grade of 7 was given being the same as 6 but with red color having permeated beyond 30 cm up into the connecting line).

**Figure 1:**
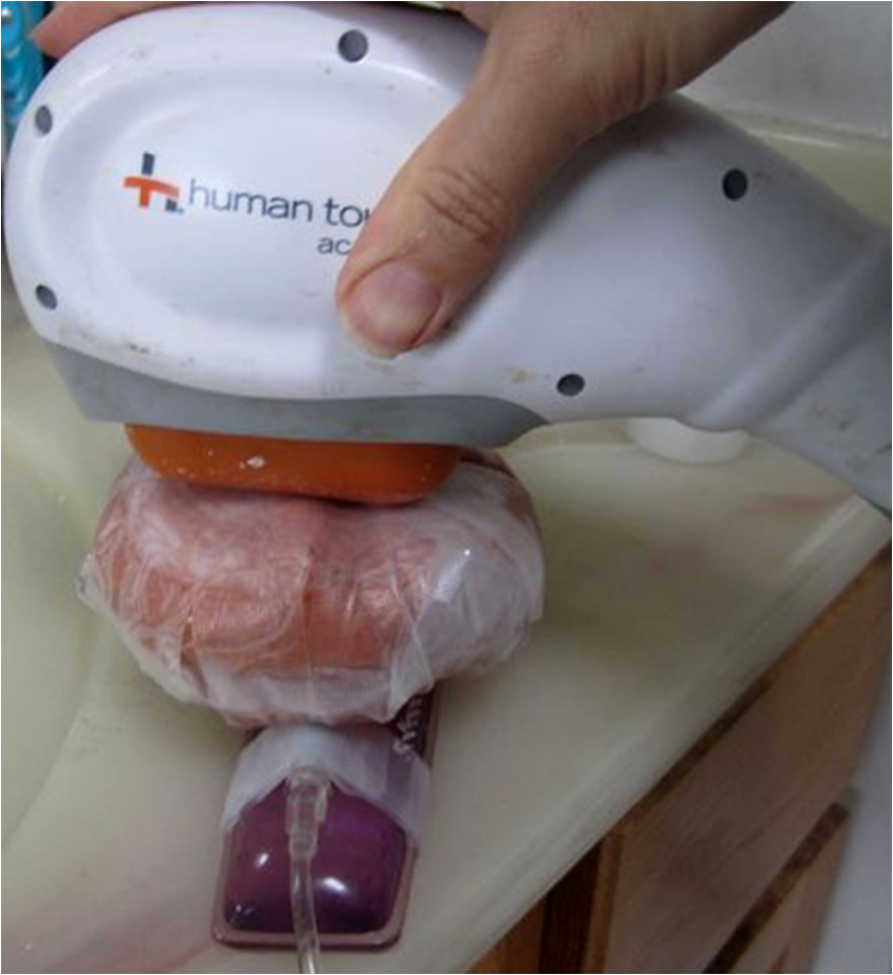
**Our vibration set up.** A 50 Hz massager was intermittently engaged upon a 4 cm thick meat slab overlying our clotted catheter segment. Visible is the pressurized connecting line protruding from underneath the slab. Not seen (under the slab) is the capped, 4 mm lumen Soft Flo Catheter segment with indwelling clot. To view the application in real time, please refer to the following internet link: http://www.youtube.com/watch?v=6CIcDttuERA.

A 4 cm thick slab of Salami meat (thickness resembling a typical distance from human chest wall to anterior surface of the heart [Author’s experience in Echocardiography]), was then placed over the clotted catheter system. The slab was covered with its original plastic wrapping and re-enforced with tape (again Transpore 3M) so its shape would hold during forceful LFVP application. The slab was then randomized to receive intermittent (engaged and very briefly disengaged at one second intervals) 50 Hz, ~4.0 mm LFVP, vs. no LFVP for 20 minutes. HS (in pressure sleeve) was manually pulsated from ~80 mm Hg to ~120 mm Hg at a rate co-ordinated such that the “diastolic” phase of the arterial like pulsations matched intermittent LFVP delivery. A second experimenter (pulsating the HS bag) was kept blinded to whether the vibration was actually applied to the clotted system or to a sham meat patty. LFVP transmission from slab to catheter was at all times visually verified by an obvious vibratory response of the connecting line which projected from underneath the slab (See Figure 1).

Periodic small breaks (a dozen or so seconds cessation of LFVP engagement) were taken every 5 minutes during the 20 minute treatment period so the investigator could briefly inspect the integrity of the catheter system, rest his hand, and document for any changes to the clotted system.

After 20 minutes of LFVP (vs. no LFVP) the meat slab was removed and connecting line was fully constricted. We then photographed the system to document for signs of clot disruption (i.e. clot morphology, fluid channel development along the sides or within the clot, clot mobilization, clot fragmentation, and degree of dissolved clot constituent mixing as seen as a red color having migrated within the catheter segment and if applicable up the connecting line). The catheter segment was then disconnected from the connecting line and aspirated of visibly accessible fluid via an 18 GA, 6.00 in BD spinal needle. To prevent accidental clot removal care was taken to keep the beveled opening of the needle against the catheter wall during aspirations, and aspirations were only attempted beyond a leading edge of a clot when there was a clear indentation or equivalent accessible fluid pathway to allow access without significantly disturbing the clot. In rare cases when the clot migrated proximally within the catheter segment the distal cap was first unplugged and forward flow was slowly permitted from the connecting line to bring the clot back to its original resting position, where-after the distal cap was re-secured and the catheter segment was aspirated of remaining fluid per normal. The catheter was then detached from the curved gel pad and if necessary aspirated again for any further accessible fluid which may have (after handling) permeated accessibly to the clot’s surface. An investigator then held the catheter segment proximal blue tipped end upright for 2 minutes following which (if any additional accessible fluid was seen) a third and final aspiration was performed. The catheter segment was then wiped (with particular attention to any tape residue) and re-weighted to establish final clot weight. Aspirations and post weighting were provided by the second investigator (who was blinded to the type of treatment each clot had received). Percent % dissolution of thrombus was derived as (pre clot weight – post clot weight)/pre clot weight × 100%.

In a second set of sample runs we repeated the experiment whereby a 0.5 ml bolus of SK (15,000 IU/100 microlitre) was delivered to fill the proximal, blue tipped end of our catheter segment in compliment with the adjoining distal aspect of our connecting line. This equated, depending on clot size, to about a 1.5 to 2.5 cm (most commonly ~ 2.0 cm) leading edge upstream delivery of SK with respect to the leading (interfacing) edge of the clot. Remote SK administration was intended to mimic an intravenous, systemic delivery scenario (typical of pre-hospital thrombolysis applications in STEMI) where an initial concentration of SK in direct proximity to a culprit thrombosis would of course be negligible.

### Measured parameters and statistics

Five clot disruptive parameters were noted in this experiment

1) Percent Clot Dissolution (for assessment of LFVP”s ability to de-bulk clot, with or without a thrombolytic agent). Percent Clot Dissolution was calculated as (pre clot weight – post clot weight)/pre clot weight × 100%.

2) Dissolved clot constituent mixing within the catheter segment and if applicable up the connecting line (to confirm clot dissolution, and for assessment of LFVP’s ability to provide upstream fluid turbidity to assist clot erosion and provide a facilitated diffusive mechanism for enhanced drug delivery). A “Relative Mixing Score” (+0 to +7) was calculated for each sample as the difference between the initial and maximum observed mixing scores during the treatment period.

3) Development of clot length intra-luminal fluid channels, including documentation of speed of channel development (for assessment of LFVP’s ability to induce morphologic changes in a clot within a vessel lumen consistent with an expectation for early reflow).

4) Clot mobilization, (for assessment of LFVP’s propensity to generally destabilize and clear a clot from its original resting position), and

5) Clot fragmentation (for assessment of LFVP’s propensity to generally disrupt or break apart clot).

Values for Percent Clot Dissolution and Relative Mixing Score (LFVP samples vs. controls) were computed for their Means +/− Standard Deviation, and then compared for statistical difference by the Student’s 2 – tailed T test.

Values for development of; clot length fluid channels, clot mobilization and clot fragmentation (LFVP samples vs. controls) were reported as “Yes” or “No” and then computed to a percentage and compared for statistical difference by the Fisher’s 2 – tailed exact test.

Time to first observation of clot length channel development was reported in minutes (i.e. 5, 10, 15 or 20 minutes).

*P values of* less than or equal to 0.05 were considered statistically significant.

## Results

### Pulsated HS only (without SK)

#### Percent clot dissolution

LFVP samples demonstrated a minor but consistent enhancement of Percent Clot Dissolution vs. controls with 23.0% +/− 5.0% vs. 1.8% +/− 1.2% respectively (p < 8.5 E - 6).

#### Dissolved clot constituent mixing

LFVP samples demonstrated statistically superior Relative Mixing Scores vs. controls with values of 5.0 +/− 0.5 vs. 0.8 +/− 0.5 respectively (p < 2.8 E −6). Dissolved red blood constituents fully diffused within the catheter segment and often migrated well up into the connecting line in the LFVP group, whereas this phenomenon (to this degree) was completely absent in the control group.

#### Development of clot length fluid channels

LFVP samples showed a strong superiority in development of clot length fluid channels (notably at the clot’s edges) versus the controls, with the effect exclusively occurring in the LFVP group (8/8 vs. 0/8 respectively, p < 0.0002).

Development of clot length fluid channels were observed to occur relatively late in the course of treatment, usually within the last 5 to 10 minutes of the total 20 minute LFVP period.

#### Clot mobilization and fragmentation

LFVP samples occasionally demonstrated clot mobilization (2/8) and rarely clot fragmentation (1/8) which again occurred exclusively in the LFVP group. These results however did not statistically differ in comparison with the control group (p < 0.5 and p = 1.0 respectively).

Results are shown below in tabular form (see Tables 1, 2, 3, and 4).

**Table 1:** Summary of results (HS only group)

**No.**	**Donor**	**Treatment**	**Pre-Wt.**	**Post Wt.**	**(%)**	**R.M.S.**	**Channels**	**(min)**	**Mobilized**	**Fragmented**
1	HG	Control	0.357	0.353	1	+1	No	-	No	No
2	AH	Control	0.346	0.333	4	+0	No	-	No	No
3	HG	Control	0.360	0.353	2	+1	No	-	No	No
4	AH	Control	0.330	0.327	1	+1	No	-	No	No
5	HG	Vibrate	0.287	0.231	20	+5	Yes	15	Yes	No
6	AH	Vibrate	0.229	0.188	18	+5	Yes	20	No	No
7	AH	Control	0.280	0.282	0	+1	No	-	No	No
8	HG	Vibrate	0.279	0.190	32	+5	Yes	10	Yes	Yes
9	HG	Control	0.377	0.371	2	+1	No	-	No	No
10	AH	Vibrate	0.423	0.310	27	+5	Yes	20	No	No
11	AH	Control	0.290	0.285	2	+1	No	-	No	No
12	HG	Vibrate	0.331	0.268	19	+5	Yes	15	No	No
13	AH	Vibrate	0.267	0.214	20	+4	Yes	20	No	No
14	HG	Vibrate	0.311	0.246	21	+5	Yes	20	No	No
15	AH	Control	0.299	0.294	2	+0	No	-	No	No
16	HG	Vibrate	0.323	0.236	27	+6	Yes	20	No	No

Results of Percent Clot Dissolution (%), Relative Mixing Score (+ 0 to +7), clot length fluid channel development (Yes or No), time by which clot length fluid channels were first observed (min), clot mobilization (Yes or No) and clot fragmentation (Yes or No) in view of a 20 minute exposure of pulsated HS coordinated with “diastolic” timed LFVP (“Vibrate”) versus pulsated HS alone (“Control”). Donors, AH = Andrew Hoffmann (male), HG=Harjit Gill (female).

**Table 2:** Mean percent clot dissolution +/− standard deviation of samples when treated by pulsated HS and LFVP (LFVP), vs. no LFVP (Control)

**Group**	**#Samples**	**% Clot dissolution (mean +/− standard deviation)**
LFVP	8	23.0% +/− 5.0%
Control	8	1.8% +/− 1.2%

The differences were statistically significant (p < 8.5 E - 6).

**Table 3:** Percentage development of clot length fluid channels of samples when treated by pulsated HS and LFVP (LFVP), vs. no LFVP (Control)

**Group**	**#Samples**	**Development of clot length fluid channels**
LFVP	8	100%
Control	8	0%

The differences were statistically significant (p < 0.0002).

**Table 4:** **Mean relative mixing scores +/− standard deviation** of samples when **treated by pulsated HS and LFVP (LFVP), vs. no LFVP (Control)**

**Group**	**#Samples**	**Relative mixing score (mean +/− standard deviation)**
LFVP	8	5.0 +/− 0.5
Control	8	0.8 +/− 0.5

The difference was statistically significant (p < 2.8 E −6).

**Figure 2:**
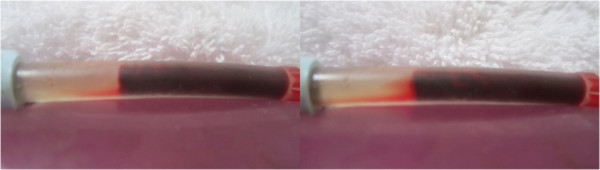
**Pre and post control sample (HS only group).** Image (left) depicts the pre-condition of a typical control sample (from sample no. 9). Image (right) depicts the sample following 20 minutes of pulsated HS only, showing only minor changes.

**Figure 3:**
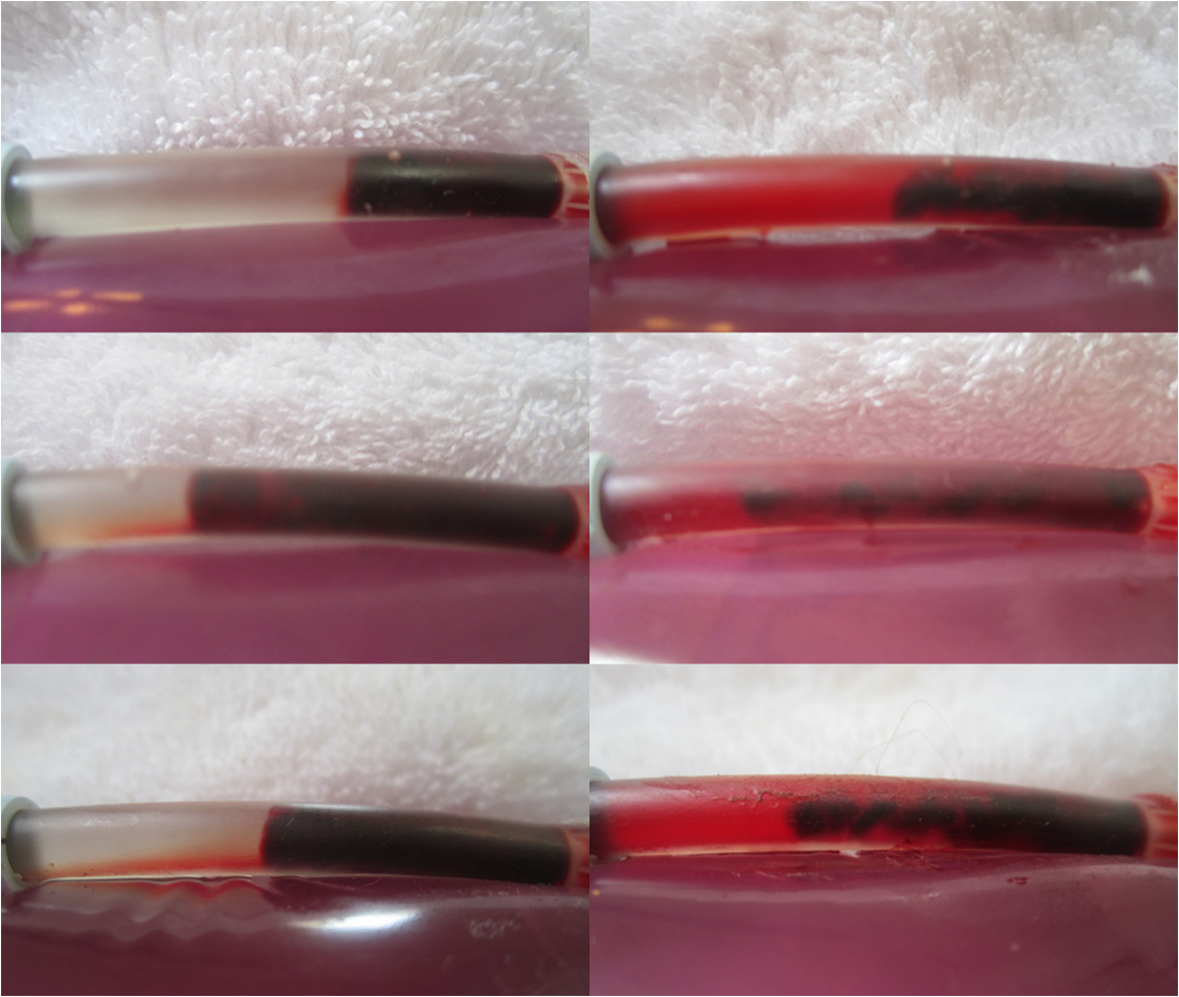
**Pre and post LFVP treated samples (HS only group).** Images (left) depict the pre-condition of three typical LFVP samples (top to bottom, from sample no.’s 6, 10 and 16 respectively). Images (right) depict the samples following 20 minutes of pulsated HS coordinated with “diastolic” timed LFVP. Note the development of clot length fluid channels and extensive mixing of red colored dissolved clot constituents within the catheter segment.

**Figure 4:**
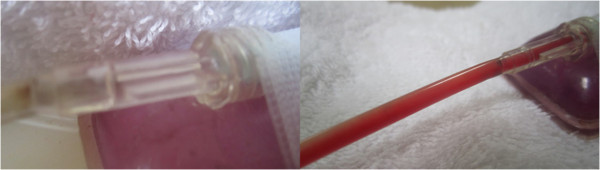
**Post treatment clot constituent mixing (Control vs. LFVP sample).** Image (left) shows the typical (and absent) degree of post treatment dissolved clot constituent mixing into the connecting line of a control sample (from sample no. 9). Control samples never at any time showed any permeation of red color into the connecting line. Image (right) shows the comparatively pronounced degree of mixing commonly observed in a LFVP sample (image taken from sample no. 10).

**Figure 5:**
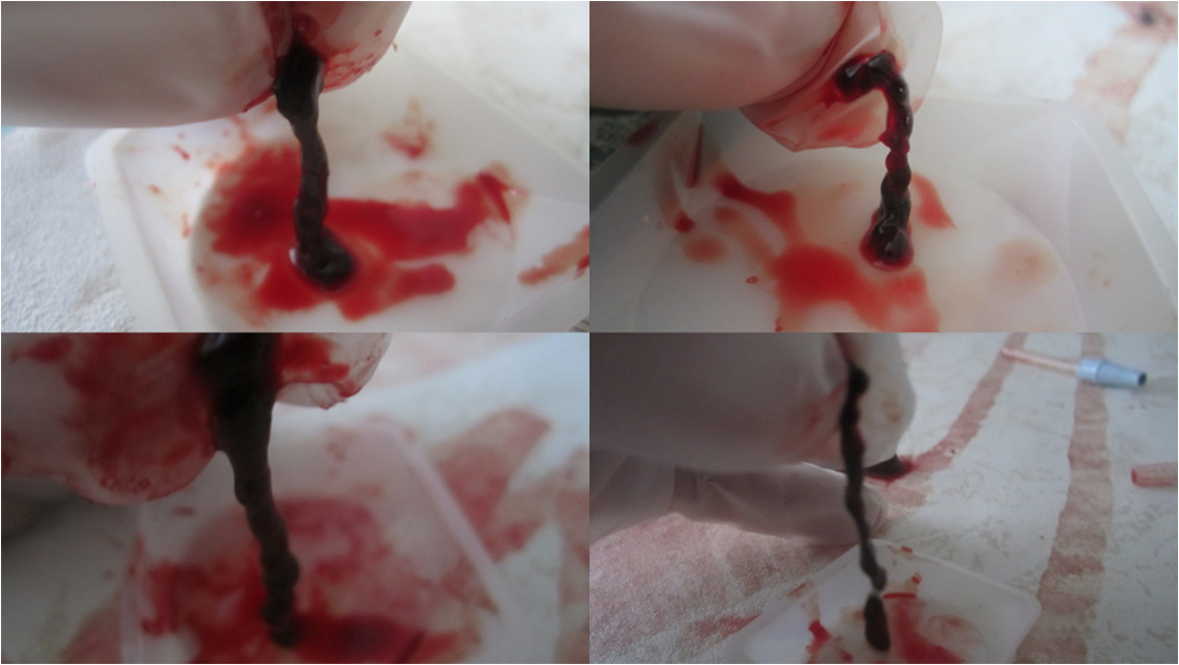
**Post treatment clot morphology (Control vs. LFVP samples).** Images (left) show the post condition of a pair of control clots following treatment (top to bottom from sample no. 11 and 9 respectively). Images (right) show the post condition of a pair of LFVP treated clots (top to bottom from sample no. 16 and 10 respectively). Note the smooth surface of the control clots in comparison to the more jagged, eroded appearance of the vibrated clots.

**Figure 6:**
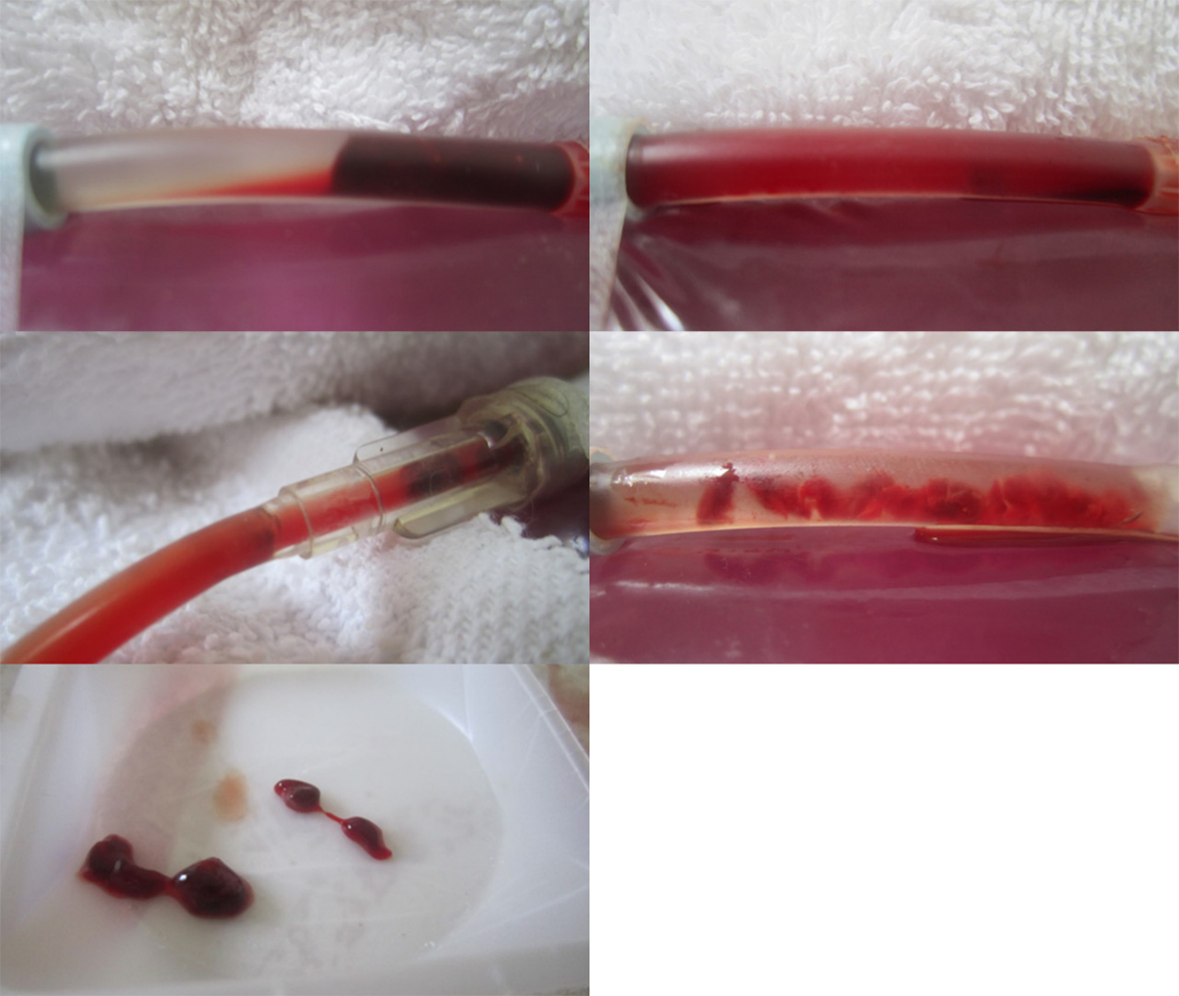
**Images from LFVP sample no. 8 which led to clot fragmentation.** Image (upper left) depicts the clot’s pre-condition (mixing score graded 0). Image (upper right) depicts the catheter segment following 20 minutes of “diastolic” timed LFVP (in this sample the clot migrated proximally from the catheter’s distal cap). Image (middle left) shows dissolved clot constituent mixing, including part of the clot, which had migrated up the connecting line (mixing score graded 5, Relative Mixing Score therefore +5). Image (middle right) shows the clot after being flowed back to its original position. Image (bottom) shows a sample of the expired clot which had fragmented.

Typical pre and post treatment conditions of control versus LFVP treated clots are shown in Figures 2, and 3 respectively. A typical degree of post treatment dissolved clot constituent mixing having permeated into the connecting line of a control vs. LFVP sample is shown in Figure 4. Typical post treatment conditions of a pair of control vs. LFVP clots (following expiration from the catheter segment) are shown in Figure 5. Photograph’s in documentation of the LFVP run which showed clot mobilization and fragmentation (regarding sample no. 8) are depicted in Figure 6.

### Pulsated HS with remotely administered SK

#### Percent clot dissolution

SK enriched LFVP samples demonstrated a marked, significant enhancement of Percent Clot Dissolution in comparison to controls with 51.0% +/− 4.6% vs. 3.0% +/− 1.5% (p < 9.8 E – 6).

The mean difference improvement in Percent Clot Dissolution in the SK enriched LFVP group (48%) versus the SK non-enriched, HS only LFVP group (21%) was more than double.

#### Dissolved clot constituent mixing

SK enriched LFVP samples continued to demonstrate statistically superior Relative Mixing Scores vs. controls with values of 5.1 +/− 0.6 vs. 0.9 +/− 0.4 respectively (p < 2.8 E-6).

SK enriched LFVP samples usually demonstrated a high degree of clot constituent mixing (i.e. up into the connecting line) relatively early during LFVP therapy, but then later during treatment the red color indicator would often dissipate within the connecting line such that it was no longer (or just barely - faintly) evident. In these cases the *maximum* degree of observed mixing over the course of therapy was taken for statistical purposes.

#### Development of clot length fluid channels

SK enriched LFVP samples continued to show a strong statistically significant superiority in development of clot length fluid channels versus the controls (8/8 vs. 0/8 respectively p < 0.0002).

Development of clot length fluid channels were observed to occur relatively early in the course of SK enriched LFVP treatment (i.e. in comparison to the HS only LFVP groups), usually within the first 5 to 10 minutes of the total 20 minute treatment period.

#### Clot mobilization and fragmentation

SK enriched control samples never demonstrated clot mobilization or fragmentation. LFVP samples with SK demonstrated clot mobilization once (1/8), and never fragmented (0/8). The differences between the test groups were not statistically significant (p = 1.0 in both cases).

Results are shown below in tabular form (see Tables 5, 6, 7, 8 below).

**Table 5:** Summary of results (SK enriched group)

**No.**	**Donor**	**Treatment**	**Lytic Dist**	**Pre-Wt.**	**Post Wt.**	**(%)**	**R.M.S.**	**Channels**	**(min)**	**Mobilized**	**Fragmented**
1	AH	Control	2.1 cm	0.258	0.254	2	+1	No	-	No	No
2	AH	Vibrate	2.0 cm	0.297	0.184	38	+5	Yes	10	No	No
3	HG	Vibrate	2.0 cm	0.295	0.177	40	+5	Yes	10	No	No
4	HG	Control	2.1 cm	0.288	0.280	3	+1	No	-	No	No
5	AH	Vibrate	1.5 cm	0.371	0.206	44	+6	Yes	10	No	No
6	AH	Vibrate	2.5 cm	0.255	0.126	51	+4^1^	Yes	5	No	No
7^2^	HG	Vibrate	1.5 cm	0.352	0.161	54	+5	Yes	10	No	No
8	HG	Control	2.3 cm	0.262	0.246	6	+0^3^	No	-	No	No
9	AH	Vibrate	2.5 cm	0.236	0.061	74	+6	Yes	5	Yes	No
10	AH	Control	1.5 cm	0.453	0.436	4	+1	No	-	No	No
11	HG	Vibrate	1.8 cm	0.303	0.125	59	+5	Yes	5	No	No
12	AH	Control	2.1 cm	0.286	0.280	2	+1	No	-	No	No
13	HG	Control	1.9 cm	0.299	0.290	3	+1	No	-	No	No
14	HG	Control	1.6 cm	0.341	0.338	1	+1	No	-	No	No
15	AH	Vibrate	2.3 cm	0.255	0.133	48	+5	Yes	5	No	No
16	HG	Control	2.3 cm	0.259	0.251	3	+1	No	-	No	No

Results of; initial lytic to clot separation distance (cm), Percent Clot Dissolution (%), Relative Mixing Score (+0 - +7), clot length fluid channel development (Yes or No), time to first observation of clot length fluid channels (min), clot mobilization (Yes or No) and clot fragmentation (Yes vs. No), in view of a 20 minute exposure of remotely administered SK in conjunction with “diastolic” timed LFVP (Vibrate) vs. no LFVP (Control). Donors, AH = Andrew Hoffmann (male), HG=Harjit Gill (female).

^1^Diminished mixing score in this sample was likely attributed to the relatively small initial clot size (with a leading edge ~2.5 cm from SK).

^2^This sample run developed a significant leak between the connecting line and blue tip connector of the catheter segment which could not be corrected. Only about 10 minutes of effective LFVP therapy was administered. Mixing score was based on maximum observed prior to the leak.

^3^Relatively low mixing score in this sample may have also been attributed to a relatively small clot size (with leading edge 2.3 cm from SK).

**Table 6:** Mean percent clot dissolution +/− standard deviation of samples when treated by pulsated HS-SK and LFVP (LFVP - SK), vs. no LFVP (Control - SK)

**Group**	**#Samples**	**% clot dissolution (mean +/− standard deviation)**
LFVP – SK	8	51.0 % +/− 4.6%
Control – SK	8	3.0 % +/− 1.5%

The differences were statistically significant (p < 9.8 E −6).

**Table 7:** Percentage development of clot length fluid channels of samples when treated by pulsated HS-SK and LFVP (LFVP - SK), vs. no LFVP (Control - SK)

**Group**	**#Samples**	**Development of clot length fluid channels**
LFVP - SK	8	100%
Control - SK	8	0%

The differences are statistically significant (p < 0.0002).

**Table 8:** Mean relative mixing scores +/− standard deviation of samples when treated by pulsated HS - SK and LFVP (LFVP - SK), vs. no LFVP (Control - SK)

**Group**	**#Samples**	**Relative mixing score (mean +/− standard deviation)**
LFVP - SK	8	5.1 +/− 0.6
Control - SK	8	0.9 +/− 0.4

The difference was statistically significant (p < 2.8 E −6).

**Figure 7:**
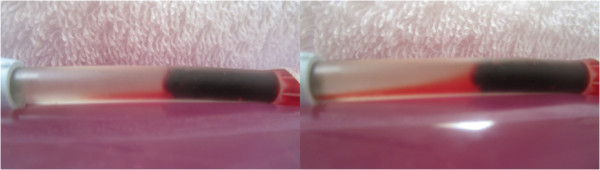
**Pre and post control sample (SK enriched group).** Image (left) depicts the pre-condition of a typical SK enriched control sample (from sample no. 1). Image (right) depicts the sample following 20 minutes of pulsated fluid only showing only minor changes.

**Figure 8:**
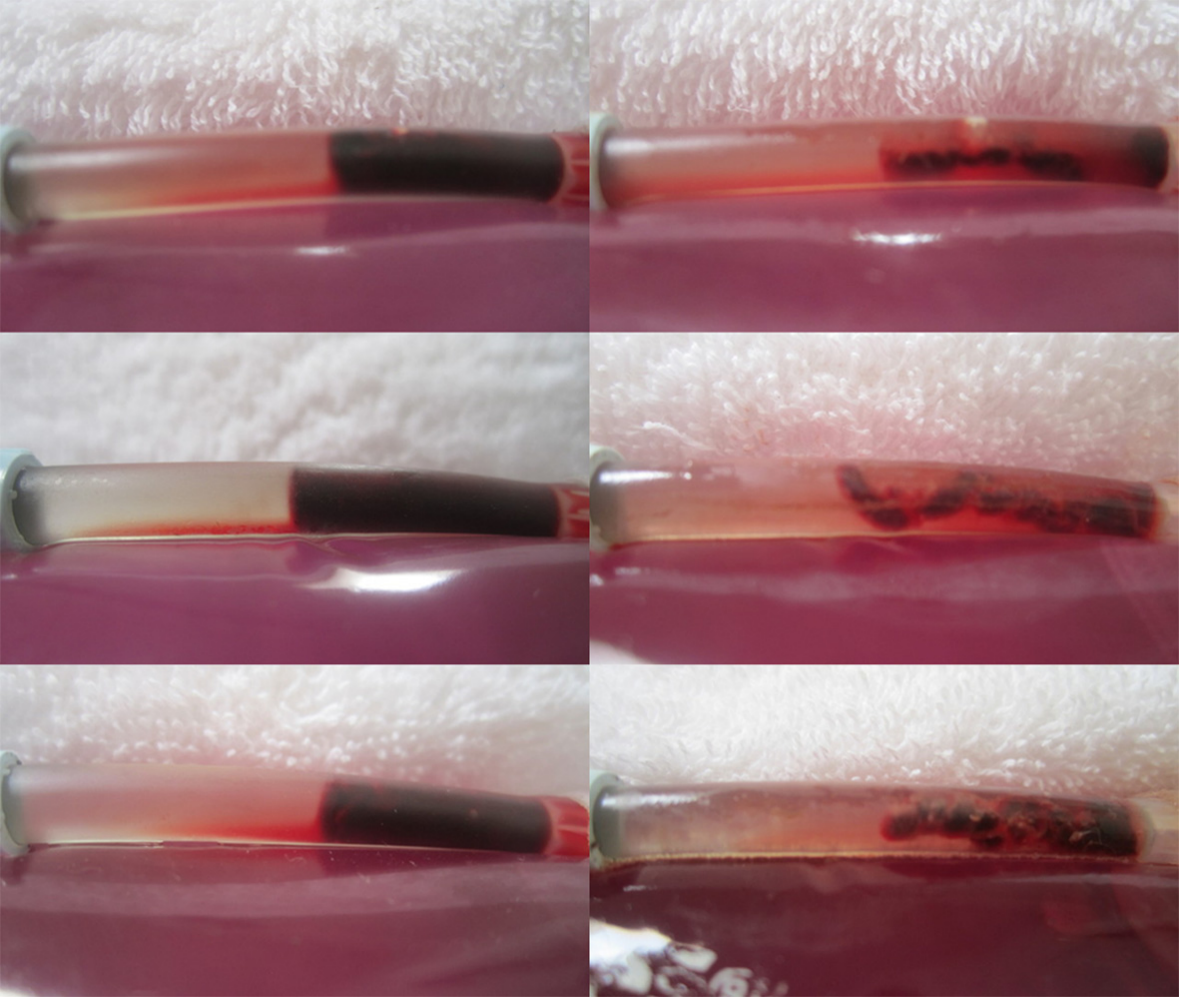
**Pre and post LFVP treated samples (SK enriched group).** Images (left) depict the pre-condition of three SK enriched LFVP samples (images, top to bottom taken from sample no.’s 6, 11 and 15 respectively). Images (right) depict the samples following 20 minutes of pulsated fluid coordinated with “diastolic” timed LFVP. Note the obvious decrease in clot size and development of substantial clot length fluid channels within the catheter segment.

**Figure 9:**
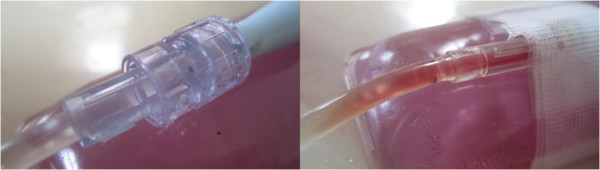
**Post SK treated clot constituent mixing (Control vs. LFVP sample).** Image (left) shows the typical (and again absent) degree of post treatment dissolved clot constituent mixing into the connecting line in a SK enriched control sample (from sample no. 1). Image (right) shows the comparatively pronounced mixing observed during an SK enriched LFVP application (from sample no. 15). Note the relative faintness of red color in the LFVP SK treated sample which we attributed to a lytic induced dissipation effect.

**Figure 10:**
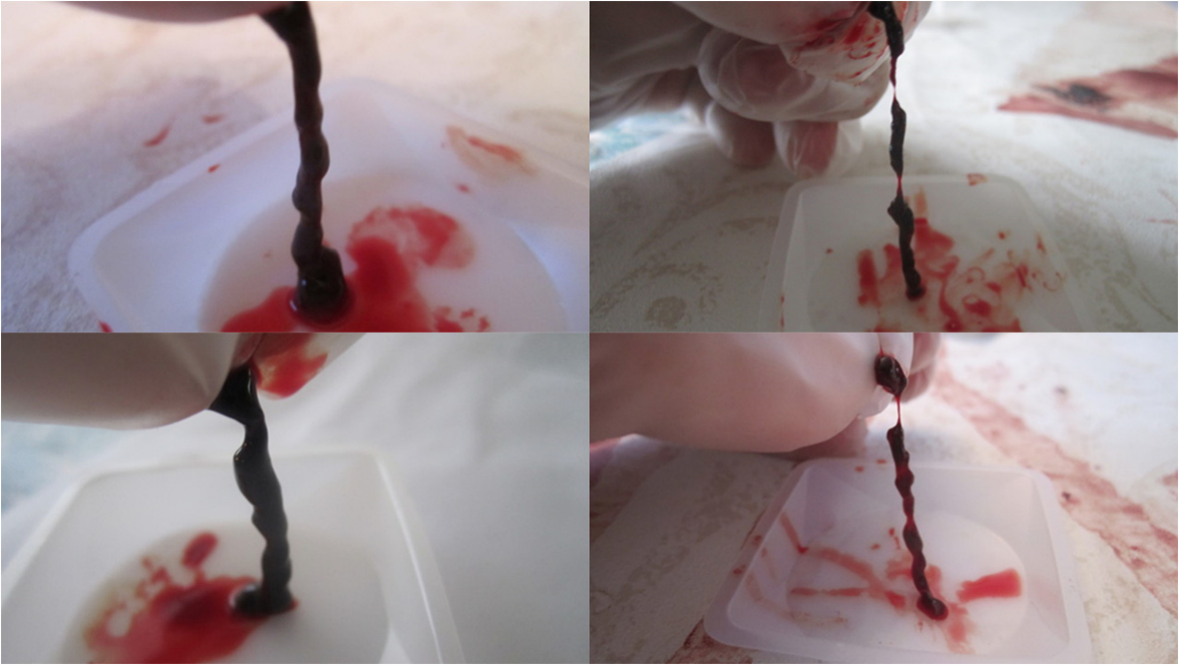
**Post SK treated clot morphology (Control vs. LFVP samples).** Images (left) show the post condition of a pair of SK enriched control clots following 20 minutes of pulsated fluid only (top to bottom from sample no. 4 and 1 respectively). Images (right) show the post condition of a pair of SK enriched LFVP treated clots (top to bottom from sample no. 7 and 11 respectively). Note the relatively smooth surface of the control clots in comparison to the much more eroded appearance of the vibrated clots which are held intact by tiny strands.

**Figure 11:**
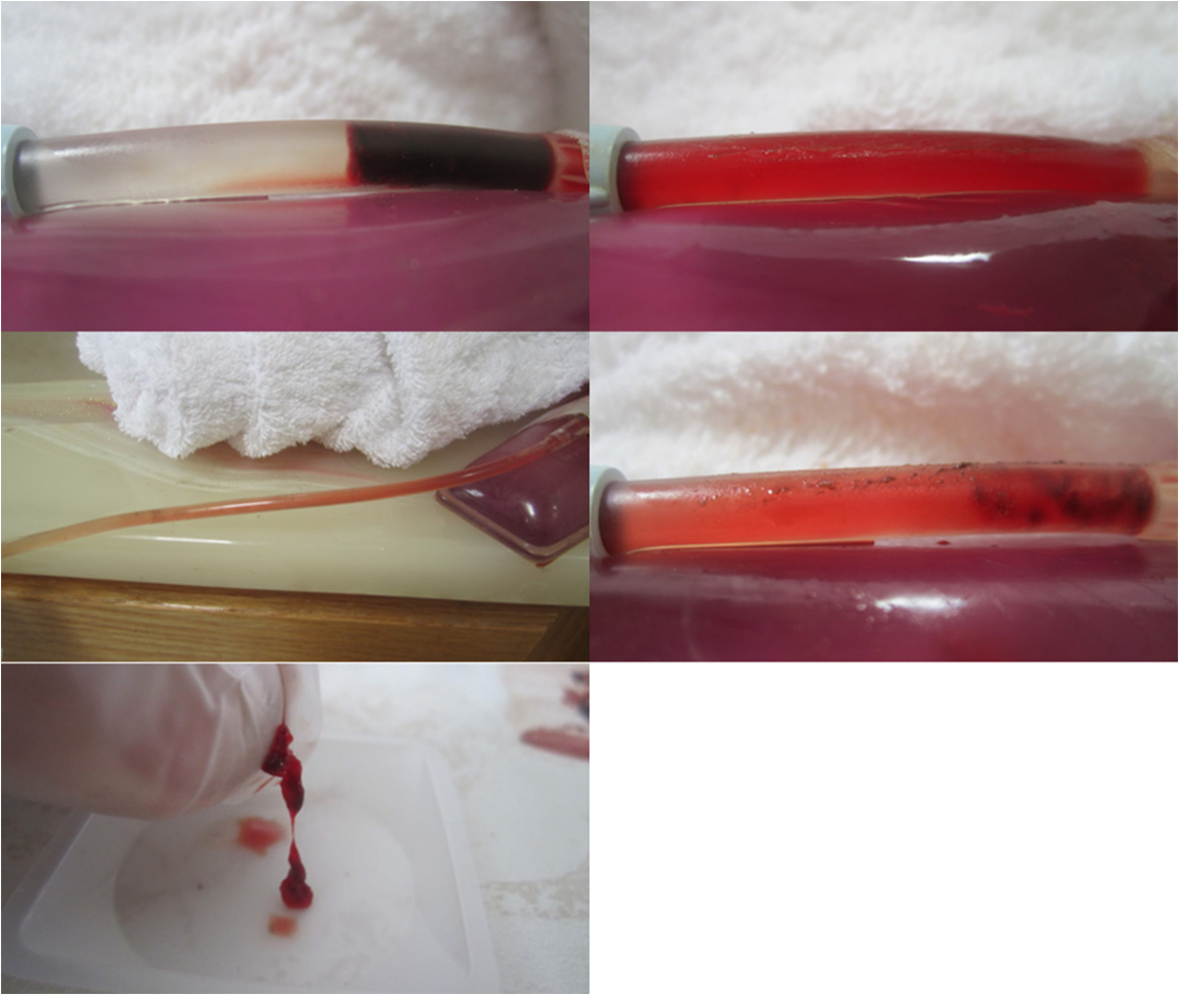
**Images of SK enriched LFVP treated sample no. 9 yielding clot mobilization.** Image (upper left) depicts the clot’s pre-condition with SK administered 2.5 cm from the clot interface (mixing score graded 0). Image (upper right) depicts the catheter segment following 20 minutes of “diastolic” timed LFVP. In this sample the clot migrated proximally and is hidden within the catheter’s blue connecting tip. Image (middle left) shows dissolved clot constituent mixing up into the connecting line (mixing score graded 6, Relative Mixing Score therefore +6). Image (middle right) shows the clot after being flowed back to its original position. Image (bottom) shows the collected clot sample which had not fragmented.

Typical pre and post treatment conditions of clots exposed to pulsated HS with remotely administered SK without and with “diastolic” timed LFVP are shown in Figures 7 and 8 respectively. The degree of dissolved clot constituents permeating up into the connecting line of a typical SK enriched control versus LFVP sample is shown in Figure 9. The post treatment condition of a pair of SK enriched control vs. LFVP treated clots (following expiration from the catheter segment) are shown in Figure 10. Photographs in documentation of the SK enriched LFVP run which caused clot mobilization (regarding sample no. 9) are shown in Figure 11.

## Discussion

This experiment confirms that LFVP (50 Hz, ~4.0 mm), periodically applied during the diastolic phase of a series of arterial like pressure pulses and engaged upon a 4 cm meat barrier provides enhanced clot disruptive effects in an underlying coronary like tube system. This is a first demonstration that LFVP engaged across a chest wall sized barrier can penetrate to yield potential thrombo-clearing effects, with the effects accentuated in conjunction with a remotely delivered thrombolytic agent.

The first notable finding in our experiment was an obvious development of clot length fluid channels in all LFVP samples (i.e. SK enriched and non-enriched) which were completely absent in all control samples. Such channeling alongside or within a clot would certainly predict a therapeutic benefit as early reflow along with enhanced penetration of systemically delivered therapeutic drug agents would undoubtedly be promoted. As LFVP is known to produce convective currents [33,34], it is probable that LFVP induced shear forces within the HS (+/− SK) solution likely acted upon (or eroded) the loosely aggregated platelet surface along the young clot’s edges. It is also possible that vibro-agitation with accompanying catheter wall deformations may have added to the erosive effect, and at least partly caused the clot to unfurl, or lay more extended lengthwise along the catheter’s lumen (which was a general observation noted in our experiment). Channel development generally occurred faster and more extensively in the SK enriched vs. non-enriched group.

A second notable finding was that while we observed a consistent but somewhat “under-whelming” improvement in Percent Clot Dissolution in the non-SK enriched LFVP group versus control (i.e. 21% - which might be to slight a change to predict a clinical benefit), we however more than doubled this improvement (to 48%) in the SK enriched group. The improved effectiveness of LFVP with remotely administered SK (a pairing which we have dubbed “*Vibrinolytic Therapy*”) is likely explained by a combination of introduced proximal fluid turbulent mixing (as a means for enhanced thrombolytic delivery - discussed more thoroughly below), along with the above mentioned development of clot length fluid channels which would enhance clot surface area exposure and fibrin binding sites to the penetrating SK molecule.

A third notable finding was an observed marked enhancement of dissolved clot constituent mixing in the LFVP samples (as evidenced by the diffusive permeation of partially transparent red color within the full height of the catheter segment and up into the connecting line), which was substantially unobserved in the control samples. This finding, besides confirming that significant clot dissolution had taken place, supports our suspicions that LFVP may have promoted delivery of SK towards the clot interface by a turbulent mixing phenomenon. Indeed, the relatively slow mass transport of systemically introduced clot busters down occluded thrombosed arteries and into clots (where the process is by in large dictated by arterial based filtration pressure with non-facilitated diffusion [35]) has certainly been an Achilles Heel in the mechanistic effectiveness of conventional, passively introduced IV thrombolytic therapy. Clearly that remotely delivered SK in our experiment was shown to be negligibly effective in clot dissolution without LFVP and note-ably effective with LFVP supports the view that a degree of enhanced drug transport had most probably taken place.

That LFVP enhances mixing between two adjacent fluids is supported in fluid mechanics. It has been solidly established that mix ability of solutes and parallel fluids within an agitated fluid system is several orders of magnitude greater than what is seen in laminar (and especially absent) flow, due to the introduced random velocity and density gradients which cause eddies and vortices which greatly accelerate diffusion and mass transport [36,37]. Indeed the correlation of increased mass transfer co-efficient between two fluids given an added LFVP application has been experimentally verified by Hancil et al. [38]. Further, Oberti et al., have shown enhanced mixing of particulate laden fluids in articulating channels (analogous to articulating blood vessels) secondary to external LFVP [39,40]. Accordingly, LFVP mixing devices such as produced by Resodyn™ Acoustic Mixers (operable at a nominal frequency of 60 Hz) have found common use in industrial mixing of both fluids and solids.

A fourth notable finding in our experiment was that clot mobilization within the catheter segment was quite rare, and when occurring was also only observed in an LFVP group. While the mechanism for this is speculative, it is likely that the transmitted vibration simply caused disadhearment (or detachment) of the clot from along the catheter walls and the distal cap, which thereby allowed the clot to move freely within the agitated solution. There was no statistical difference in the likelihood of mobilization between SK enriched (1/8) and non-enriched (2/8) LFVP treatment groups.

A final notable finding to our experiment was that clot fragmentation (with division of clot into separated pieces) was also very rare, occurring only once in a SK non-enriched LFVP “HS only” group. Again while the mechanism for this is also speculative, it is likely that clot fragmentation may be simply a result of more extensive clot erosion with possibly an increased surface tension along the clot’s length during vibration induced lengthening and de-furlment. Inspection of LFVP treated clots (although more commonly in the SK enriched samples) did show aspects of extreme clot thinning into tiny strands – which amazingly were able to keep the clot intact. It is worth mentioning how strong these tiny strands (which are often as thin as a strand of spider’s web!) can be, being able to support the majority weight of the remainder of a clot when being held upright by the hand of an investigator (refer to Figure 10).

Our findings raise the interesting question whether chest wall delivered LFVP in the low sonic frequency ranges (as opposed to, or maybe even working co-operatively with therapeutic ultrasound?) may hold potential as a knock out “one–two punch” in aid of clot disruptive, and more particularly IV thrombolytic therapy in treatment of STEMI. First by mechanically eroding clot surface layer and altering clot morphology to promote intra-luminal fluid channel development (which would enhance early flow and uncover fibrin binding sites), and second by facilitating diffusive bulk flow of clot disruptive drug agents from a concentration rich systemic circulation towards a concentration poor, otherwise stagnant, thrombosed coronary vessel.

It should be pointed out that transthoracic LFVP is prophesized to promote early clearance of acute coronary thrombosis by numerous additional mechanisms, which are beyond the scope of our present study.

First, it has been shown both clinically and in animal models that dLFVP when applied to the heart improves ischemic LV function, by improved myocardial relaxation, which has been attributed to enhanced diastolic filling leading to increased stroke volume by the Frank Starling mechanism [21,28,29]. This, by stabilizing a STEMI patient’s blood pressure (for example during heart failure or cardiogenic shock), would also augment systemic filtration pressures in assistance of bulk flow permeation of clot busters into a site of culprit thrombosis.

Second, improved myocardial relaxation by dLFVP would theoretically decrease coronary arteriolar and capillary flow resistance (by reduced diastolic myocardial compression) and is known to lower left ventricular (LV) diastolic pressures [21], which in combination should assist coronary flow and microcirculation during or following reperfusion.

Third, there is good evidence that LFVP carries potent vasodilatory capabilities for arteries in states of heightened vascular tone (i.e. by induced relaxation of the smooth muscles in the vessel wall) [41-43], and thus holds potential to induce direct coronary dilation at the site of acute coronary thrombosis, which often has (in at least 50% of STEMI cases) a degree of associated localized coronary spasm [44-47]. It is also worth mentioning that cyclic stress and strain exerted on an endothelial lining of an artery (which would likely be induced through a prophesized LFVP application)^c^, is predictive to cause liberation of beneficial mediators such as Nitric Oxide (NO) which is also a potent vasodilator [48]. Indeed low frequency vibration stimuli has been experimentally shown to trigger NO release in various tissues [49-51]. LFVP’s vasodilatory mechanism in particular may therefore offer a degree of near immediate early re-flow in a high percentage of STEMI victims.

Fourth, LFVP should encourage disadhearement of acute thrombosis from the coronary wall (i.e. from site of ruptured plaque), and thereby promote very early recanlization of the major culprit epicardial vessel. External LFVP’s ability to clear clot from a stenosis site has been shown previously by our group [20], and has also been demonstrated by Folts et al. in reliable and immediate clearance of acute coronary and carotid thrombosis (albeit at a lower frequency, via direct hand tapping or shaking of the acutely thrombosed vessel) in open animal models [19].

Fifth, LFVP is known to stimulate endogenous liberation of fibrinolytic mediators [52,53], thereby potentially offering a natural augmentation for localized fibrinolyis.

Six, LVFP should curtail stagnant arteriolar/capillary flow by prophylactic agitation of distal clotted fragments and by possibly lowering blood viscosity [54,55], which may further assist in microcirculation during or following reperfusion. Researchers at Mt. Sinai Medical Centre (Miami, Florida) have recently studied the effects of low sonic vibration applied to the chest wall of rats which showed an up-regulation of endothelial derived NO Synthase (eNOS) with enhanced NO release, which in addition to its therapeutic vasodilatory properties has been discussed as a potential cardio protective mechanism in limitation of ischemic reperfusion injury [56].

There were several limitations to this present study. Firstly, the degree of removal of accessible fluid by our aspiration weighting technique could not be made identical between test runs. Great care however was taken to remove fluid using the same technique and in near identical fashion between samples, and the individual providing the post weighting was kept blinded to the form of treatment the clot was to, or had received. Secondly the meat slab was only a crude approximation of an overlying chest wall (lacking muscle fibers, ligaments, fat, fibrous pericardium, pericardial fluid and lung), and the underlying clotted vessel comprised a flexible catheter segment rather than a live coronary artery. However we felt that as an early experiment to assess LFVP’s ability to transmit and provide a basic clot disruptive effect over a thick chest wall sized attenuation barrier, this would at least provide a good “go–no go” hypothesis in that if LFVP produced a negligible effect the application would most certainly fail under less ideal clinical circumstances. It should be mentioned that LFVP has been shown to penetrate effectively from the human chest wall to the heart (even causing vibratory deformations to the deep posterior wall) by Koiwa and his associates by use of a more gentle vibration device (2 mm amplitude) than what we used in our experiment [21,31]. Thirdly we had the advantage of localizing the vibrator placement upon the meat slab to ensure that the catheter segment underneath was vibrating, whereby this advantage would be absent clinically. However, the design of a vibratory attachment interface specific for inter-ribspace chest wall applications is presently underway which enables enough coverage over the base of the heart to maximize the chances that an LFVP source would generally overly a culprit coronary vessel [57]. Fourthly, we cannot say by direct observation whether the relative improvement of remotely administered SK effectiveness with LFVP was due to a process of enhanced drug delivery (by facilitated diffusion), or simply an enhanced localized interaction of thrombolytic agent at the clot interface. The minimal degree of SK clot dissolution without LFVP however (virtually absent, only 3%) strongly suggests that the controls had very little if any contact with the SK molecule, so there very likely was at least a degree of enhanced drug delivery in the LFVP samples. Finally this study, because it was not a “flow model”, provided little information on whether LFVP may promote macro-embolization of clotted fragments downstream (or more worrisome upstream, especially in view of LFVP enhanced turbidity) which has been a concern to some colleagues. We can say however that while the remnant clots following LFVP treatment had always become thin and eroded in appearance, in only one rare occurrence (in 1 of 16 samples) was actual clot fragmentation observed (see Figure 6). Hence the chances of a macro-embolic proximally situated piece of clot *breaking off* and flying out towards the systemic circulation would seem, at least from our study, unlikely. Clot fragments of course by common wisdom would generally tend to mobilize (if at all) downstream along with the vessels pressure gradient – to less harmful territory. In our experiment we did see LFVP promoted red color (representing dissolved, disaggregated, clot constituents) moving upstream towards and into the connecting line, and occasionally (in 3 of 16) samples there was a degree of upstream mobilization of the clot itself. However we point out that our system would not allow for anything *but* proximal clot mobilization (as distal flow was always blocked by the catheter segment’s distal cap), so no correlation can be drawn from our study whether there is an adverse *tendency* for LFVP to mobilize clot’s proximally versus more beneficially distally. Never-the-less LFVP induced proximal fluid turbidity warrants caution towards the potential of upstream liberation of coronary originated thrombo-emboli, hence any in- vivo study involving this prophesized application would require vigilant monitoring for stroke and acute peripheral arterial occlusion during safety analysis.

It is worth additional historical comment that our group has been placing faith in animal and human data from Koiwa and his associates (studies almost two decades old!) as gospel that dLFVP when applied to a human chest wall surface is sufficiently penetrative and causes enhanced Left Ventricular (LV) function and coronary flow in the ischemic heart. Koiwa’s work was abandoned in or about 1996, perhaps because of lack of funding, or because they were targeting use of their vibration device for treatment of heart failure (which probably would have been of little practical value).

### Future work

#### The search for ideal LFVP parameters

It should be emphasized that our choice of studying “50 Hz” (versus any other low sonic or high infrasonic frequency) was based primarily on third party reports (Koiwa et al’s work) which attest to the therapeutic effect of diastolic timed 50 Hz on LV function and ability to enhance coronary flow [28-32]. Alternatively, we have found that *100 Hz*, 0.5 mm LFVP offers clot clearing effects across a small 2 cm meat barrier [20], and Wobser et al. found that LFVP in the *50500 Hz* range disrupted big blood coagula in the stomach, with better efficacy at higher frequency levels [18]. The ability for the heart to transmit received LFVP signals transversely (i.e. along arteries and the epi-myocardium) has been reported to generally occur at frequencies in the range of 20–120 Hz [23-26], so it is conceivable that any frequency within this range may comprise a reasonable choice in causing a therapeutic agitative effect to a coronary circulation. There is also the question about vibratory pattern. Engineers at Simon Fraser University have postulated that random or swept LFVP within a select frequency range may offer superior clot disruptive effects, such as to optimize turbulence, and ensure occasional striking of an optimal resonant clot disruptive excitation or cardiac frequency [58].

It is also possible that LFVP could be combined with therapeutic ultrasound with or without co-administration of ultrasonically active IV micro-bubbles (which oscillate in response to ultrasound, to augment acoustic agitation and intra-luminal sheer forces). Indeed this could be accomplished easily by simply mounting a therapeutic ultrasound transducer as a percussive contact (with or without an accompanying imaging system) upon an active end of a low frequency sonic actuator. In such an application it could be envisioned that LFVP would provide clot erosion and vasodilatory mechanisms to promote initial recanalization of a TIMI 0 flow vessel, which would thereby assist entry of systemic micro-bubbles (as well as thrombolytic drug agents), into a thrombosed culprit circulation. Much work in deciphering an optimal frequency, vibratory pattern, and potential use of multi frequency acoustic waveforms and adjunctive intravenous agents remains in development of this field.

#### Other applications

The potential for external LFVP in the clearance of acute arterial thrombosis is far reaching beyond coronary syndromes. For example a more gentle LFVP application (i.e. to the cranium and/or neck of a patient) may be useful to expedite localized IV drug effectiveness in treatment of Acute Ischemic Stroke (AIS). In fact, Antic and his associates have demonstrated that headphone applied music (a gentle form of LFVP) enhances culprit Middle Cerebral Artery (MCA) blood flow in non thrombolysed AIS victims within a day following admission [59]. External LFVP massage could also foresee ably be utilized in the acute emergency treatment of acute pulmonary emboli (particularly in saddle emboli – a life threatening condition) or acute peripheral arterial thrombosis as an alternative or bridge to emergency surgery or catheter based removal techniques. Clinical trials would be required on all these fronts.

## Conclusion

LFVP (50 Hz, 4 mm) timed to the diastolic phase of arterial like pressure pulses and engaged upon a 4 cm thick meat slab (analogous to a chest wall barrier, overlying the heart) provides clot disruptive effects in an underlying coronary like tube system. Percent clot dissolution and speed of development of clot length fluid channels were relatively accentuated by LFVP with SK administered ~ 2 cm upstream from the clot. Transthoracic, diastolic timed LFVP warrants further study in vivo as a potential emergency treatment for STEMI.

## Endnotes

^a^An SK concentration of 15,000 IU in 100 microlitres is within the range of doses shown to have good thrombolytic effectiveness when applied in direct contact to fresh clots in quiescent states in comparable in-vitro thrombolytic testing models [60].

^b^Blood analysis of the Author’s showed normal values for CBC, PT and PTT.

^c^As LFVP is characterized by rapidly changing compressive and expansive mechanical forces in tissue, it is reasonable to postulate that LFVP would most likely expose the fluid and endothelial cells within the vasculature to such mechanical stimuli (i.e. cyclic stress and strain). Indeed, hydrodynamic analysis indicates that shear stress at the wall of vessels (including the coronaries) are significantly increased during bodily exposure to low frequency vibration in the 40 to 50 Hz range [61], hence the triggering of NO release by LFVP can therefore be hypothesized.

## Competing interest

This study was sponsored by Ahof Biophysical Systems Inc., of which AH is a director and share holder. ABS Inc. is in partnership with Simon Fraser University in development of vibrating systems to improve blood flow. For further information on investment or research partnership with regards to this technology please contact the University Industry Liaison Office of Simon Fraser University (Burnaby, B.C. Canada). The Author’s declare that they have no further competing interests.

## Authors' contributions

AH carried out phlebotomy, initial clot preparation, and vibration applications, whereby HG performed diastolic timed fluid pulsations and post treatment clot manipulations. Both author’s contributed in the design of the experiment and drafting the manuscript. Both authors read and approved the final manuscript.
